# Detection of Anomalous Behavior in Modern Smartphones Using Software Sensor-Based Data

**DOI:** 10.3390/s20102768

**Published:** 2020-05-13

**Authors:** Victor Vlădăreanu, Valentin-Gabriel Voiculescu, Vlad-Alexandru Grosu, Luige Vlădăreanu, Ana-Maria Travediu, Hao Yan, Hongbo Wang, Laura Ruse

**Affiliations:** 1 Institute of Solid Mechanics of the Romanian Academy, 010141 Bucharest, Romania; victor.vladareanu@imsar.ro (V.V.); ana.travediu@imsar.ro (A.-M.T.); 2 Faculty of Electronics, Telecommunications and Information Technology, University Politehnica of Bucharest, 061071 Bucharest, Romania; valentin.voiculescu@upb.ro (V.-G.V.); crisvlad74@gmail.com (V.-A.G.); 3 Parallel Robot and Mechatronic System Laboratory of Hebei Province, Yanshan University, Qinhuangdao 066004, China; yh@stumail.ysu.edu.cn (H.Y.); hongbo_w@ysu.edu.cn (H.W.); 4 Faculty of Automatic Control and Computers, University Politehnica of Bucharest, 060042 Bucharest, Romania; laura.ruse@cs.pub.ro

**Keywords:** software sensor data, machine-learning classifier, smartphone security

## Abstract

This paper describes the steps involved in obtaining a set of relevant data sources and the accompanying method using software-based sensors to detect anomalous behavior in modern smartphones based on machine-learning classifiers. Three classes of models are investigated for classification: logistic regressions, shallow neural nets, and support vector machines. The paper details the design, implementation, and comparative evaluation of all three classes. If necessary, the approach could be extended to other computing devices, if appropriate changes were made to the software infrastructure, based upon mandatory capabilities of the underlying hardware.

## 1. Introduction

Among our gadgets, smartphones are our closest companions. They provide the primary access into the Internet and modern amenities, they hold our private data and are becoming one of the primary means of attack against the user, be it through power viruses (or other means to consume resources) or more ordinary malware menaces (calling or texting tolled numbers, install unwanted software, send the attacker private information about the device or its owner, spy on the owner using the camera or microphone, etc.). 

For our research we picked an Android smartphone over an iPhone, primarily due to the open access into its software stack. This open source stack, provided by Google for their devices via Android Open Source Project (AOSP), includes a high-level Android framework, and an open source kernel. This level of openness into the source code of the stack allows access into both public and non-public API information, allowing for the purpose of our research access into the smartphone sensors. It also consists of the basis of the infrastructure of data collection architecture (used to obtain the raw data behind the initial dataset for this paper) described in [1] with an application for malware detection [2], using measurable events collected for a set of Android applications including both samples obtained from scientists in the field (Malgenome application set described by the authors of [3]) as well as more recent Internet sources (including [4,5] and Google Play for benign Android samples).

The purpose of this study was to assess if anomalous behavior could be detected through machine-learning classifiers based on input data sources from a variety of sensors within the device. We took into consideration that the smartphone phone itself can provide a large pool of data sources about runtime behavior. Some of this data is accessible through public APIs and an additional set can become accessible by making suitable changes into the smartphone software stack. While typical developers do not have access to non-public APIs, such an approach could provide benefits for telecom or smartphone manufacturers which have access to the AOSP stack or its equivalent from the smartphone system-on-chip (SoC) manufacturers or similar providers. By adding such an application within their phone, smartphone manufacturers could increase the intrinsic value of their product. 

Many recent papers are trying to tackle the problem of detecting anomalous behavior in modern smartphones using software sensors (measurable events). For modern smartphones approaches vary but usually involve a combination of static and dynamic behavioral extraction of interesting data pertaining to sensors of the target smartphone application.

The measurable sources of data used in static analysis fall into 4 categories according to a recent study of over 80 frameworks by Bakour et al. [6]: manifest-based, code-based, semantic-based, or application metadata-based. According to the same authors, using this data requires offline processing and is prone to weakness when obfuscation techniques are employed by the developers of malicious applications. 

Recent frameworks use a combination of static [7,8,9] and dynamic [8,9] extraction of data from sensors before being processed through machine-learning techniques. SandDroid is a recent sandbox analysis project classifying Android applications on a set of features using both static and dynamic analysis [8].

Current anomaly-detection applications span a wide array of technical solutions, from traditional machine learning and statistics, to deep learning models for computer vision related tasks, with applications in virtually all fields of technology [10,11,12]. 

Gaussian Mixture Models (GMMs) are one of the traditional approaches to anomaly detection. Zong et al. [13] use a deep auto-encoder to generate a low-dimensional representation, which is further fed into a GMM for unsupervised anomaly detection in medical applications. Aljawarneh et al. [14] use a Gaussian dissimilarity measure for anomaly detection in Internet of Things (IoT) systems, while Li et al. [15] use GMM-based clustering for an application of early warning in civil aviation.

In viewing anomaly detection more as a classification problem, there are many options for the choice of classifier. Erfani et al. [16] use Support Vector Machines and Deep Belief Networks to mitigate the impact of high dimensionality in large-scale anomaly detection. Li et al. [17] use a modified Support Vector Machine (SVM) as an alternative to computer and information system security. The excellent surveys by Chalapathy [10] and Ye [11] give a detailed outlook of possible applications using deep learning and data mining solutions.

The literature involving detection of anomalies in modern smartphones usually focuses on centralized large-scale frameworks or collaborative efforts. Centralized frameworks are used for either static code analysis [7,8] or dynamic analysis using virtual machines on host systems [8], highly efficient in running Android apps in emulation. Collaborative efforts, such as Contagio [18] rely on a mix of online tools and offline community support. We considered the approach of running applications within an actual smartphone, as there are specific limitations (e.g., thermal throttling) or information (e.g., device identifiers) that do not appear in virtual machine simulation but make an impact on application behavior on real hardware (through metrics such as CPU load, network data frequency, and throughput of which we planned on measuring through our experiments). We used as starting points data acquired on actual smartphones, not via simulated/virtualized frameworks. In this way our environment also took into consideration the influence of additional constraint factors within the smartphone (such as thermal throttling, dynamic frequency changes and overall other types of computation throttling) which can modify an application resource requirements and through this means influence the collected raw data that are used as input for the machine-learning algorithms. Virtualized infrastructures do not simulate specific smartphone behavior such as thermal throttling, dynamic processor frequency changes etc.

Furthermore, the use of such methods, as described in the paper, is relatively limited in the context of smartphone technology. Most smartphone applications tend to veer towards classical anomaly detection and statistics, such as GMMs [13,14,15] or Hidden Markov Models (HMMs) [19], data mining and deterministic decision systems [20,21], or hybrid algorithms that incorporate learning classifiers (usually a version of SVMs) and belief networks [16,17,22,23]. An extensive literature review has found no comparative studies using the proposed methods on a challenging dataset, which is itself unique, as obtained through the methods described above, and very few instances of any one of the proposed methods being employed in anomaly detection on smartphones, despite their otherwise ubiquitous presence in machine-learning and anomaly-detection applications.

While all detections are aimed at malware applications, the detection itself is done through looking for patterns of anomalous behavior, as captured by the measurable sources of information (i.e., software sensors). The use of the proposed class of methods has to do with the way in which such potentially anomalous behavior could be learned, therefore it is intentionally modelled as a classification problem, rather than a standard anomaly-detection problem (for example through GMMs), due to the format of the data and the underlying assumptions of the learning model.

The choice of algorithm has very much to do with the available dataset. For the purposes of this paper, deep learning and some data mining solutions are not well suited to a small- to medium-sized dataset. In addition, GMMs are most frequently used when there is a large non-anomalous subset to train on, or when the dataset itself is imbalanced, and when it is thought that a classifier is unlikely to learn a coherent model for the positive (anomalous) samples, which is not the case here. As such, the paper investigates three types of classifiers for the machine-learning application: Logistic Regression, a shallow neural network for pattern recognition, and SVMs. The three are evaluated on several metrics, most important of which being the F1 score on the test set. The full details of the design, implementation, and evaluation of the learning algorithms are presented in their respective sections. Finally, the results show that all the three investigated algorithms perform reasonably well, with SVMs having a slight edge. 

The remainder of the paper is divided as follows: Chapter 2 outlines the acquired data and the steps taken to curate the dataset, as well as giving a brief theoretical overview of the design of the classifier learning methods involved. Chapter 3 discusses the particulars of implementation and the obtained results for each model. Chapter 4 discusses the highlighted results and attempts to draw a conclusion based on the work reported. Opportunities and directions for future research are also discussed.

## 2. Materials and Methods

**Figure 1:**
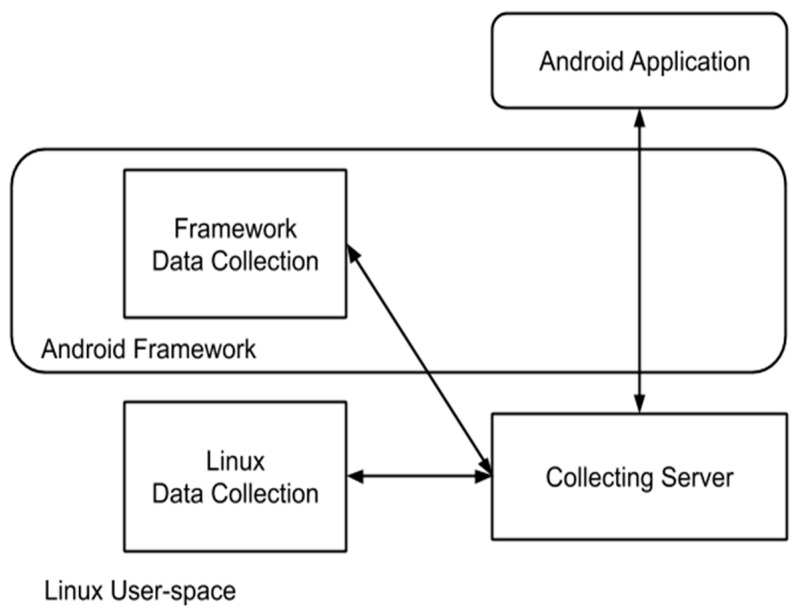
General data collection architecture.

The general architecture, described in [1] includes measurable events as sources of data, extracted with a set of data collectors [1] into a server for further processing, as shown in Figure 1. The sources of data are made available through the Android framework or Linux user-space (via procfs virtual file system entries) and are provided originally by hardware sensors or software hooks added into the framework. 

Synthesizing the information available in [1], Table 1 below shows the measurable events that were collected on a Nexus 4 smartphone running Android 4.2.2 JellyBean.

As said in [1] for the “SMS” event there are hooks for destination number and message content. For the “Call” event there is a hook for the destination number. For “WiFi”, “Camera”, “NFC”, as well as “Bluetooth” we get information about sensor activity (turning on/off). “Sensors” offer information about what peripherals are registered as sensors in the Android framework terminology as well as real-time data from them. “Camera” indicates if the application wishes to access the camera peripheral or photo content within the phone.

Through the data acquisition procedure, the smartphone remained mostly stationary, in an artificially lit room with weak GPS reception. As such, the device was not able to offer pertinent GPS or other motion (accelerometer, gyroscope, magnetometer) or lighting (ambient light) information for our experiments. We chose not to include in our smartphone a functionality (normally missing from commercial devices) that artificially injects GPS data, considering that by itself any additional GPS data injection component would represent an example of atypical application (hard to install by regular users) and behavior (and also being potentially hazardous if requiring administrative privileges into the phone in order to operate). For our research we focused on the scenarios that for malware applications the abnormal behavior would manifest primarily through disguising themselves as top (or banking) applications (for the purpose of extracting confidential login data) or by sending phone information to toll numbers or various URLs. 

Another reason not to include GPS information was that for the majority of applications in our pool this information was not required, the main part of the experiments not including applications focused on user movement information (whether it is from GPS or other types of movement based sensors).

“App Install” detects the installation of a new application within the smartphone and it offers the app name for it. “Activity” intercepts information regarding the state of the Android application: “Create”, “Start”, “Stop”, “Pause”, “Resume” and “Destroy”. The “Runtime Crash” detects the similarly named event at runtime. The “ANR” intercepts “Application Not Responding” type events generated by the Android framework following an application entering a freezing/non-functioning state. These hooks were implemented in Java and they offer the ability to acquire data for the data collection server upon request, for the purpose of detecting anomalous behavior.

Due to the user experience oriented programming in modern smartphone apps, each feature is accessible within a short number of Graphical User Interface (GUI) interactions (for example in email apps you have login, message lists, folders/categories, individual email widows and a limited set of clicking and scrolling to do in each such window), in our case exposing behavior coded within Android applications to our software sensors. Empirically, as a trade-off between time spent collecting data for each target application and the implication on overall collecting time spent on the entire application pool we studied and collected data for, we found that approximately 5 min was considered enough to stop acquiring new types of events, and thus new type of behavior, from the application.

The final application set comprises 361 Android applications; however not all of them were installed at once, but consecutively during the experiments. For each sample application, during the dataset acquisition the procedure involved side-loading (installing Android applications in apk format via the adb interface), starting the sample application and monitoring its behavior while interacting with it manually (in foreground/background) for a short period of time (approximately 5 min), then uninstalling it.

The initial data was collected through the framework described in [1,2] which relies on a data collection framework within the smartphone and the installation of an additional application that performs the real-time monitoring while running in background (called AMDS in [2]). The application pool was built by using both reputable applications (the ones that the phone came with, top Google Play applications, or similar stores for other markets such as SlideMe [4]) as well as known malware applications. The data acquisition process involved installing new applications one by one, manually interacting with each in application-specific manner (e.g., clicking buttons, swiping) while also toggling between foreground/background for the duration of the experiment (around 5 min for each app). After this step the target application was uninstalled, to prevent both reaching lack of storage space during the experiment as well as preventing some applications from influencing one another and the next target application from the list was installed and experimented with. During the training phase a data of monitoring each target application was stored locally in the phone, and during testing phase a different pool of applications was used, each application installed, monitored, local data stored updated, while the real-time monitoring application described at large in [2], was running in background, and if abnormal behavior was detected this application would be prevented from running, it would be flagged to the user who would make the final decision (to continue preventing the application from running or allow it to continue) on a case by case basis.

The raw data obtained from the experimentation is organized as a set of unique application names, a set of labels for each application (clean or malware) and 19 sets of feature data, one for each investigated feature. Inside the feature data, there are multiple values for the same {application name; feature} pair, due to multiple sensor interrogations for the same feature on the particular application. Table 2 shows an example of the raw data obtained from the sensors.

Table 3 shows the investigated features for which sensor triggers were programmed and a short description, as detailed in [1,2].

We considered the possibility that while there is a usual correlation between the number of packets and bytes transferred via WiFi, in the case of applications disguising themselves as another application, there may be a difference in communication protocols between the fake and original application that could influence the correlation between number of packets and bytes transferred. Generally speaking, even if the two features were to be correlated, the presence of two correlated features among a total of 19 would not adversely affect the learning process to any significant extent. In this particular case, however, the possible lack of correlation could, potentially, in itself be a valuable learnable pattern for detecting behavior. Therefore, it was believed that including both features would result in new information being available to the model.

The measurable events pool provided by the infrastructure described in detail in [1] and used through the monitoring application described in [2] includes events collectable from Android (e.g., SMS, Bluetooth, Call etc.), events collectable from Linux user-space (e.g., via procfs, including CPU load, memory load etc.) and there were non-numerical metrics (e.g., app name, version) which were eliminated when we curated the data set. Features for which data was not available in all instances were then eliminated to preserve the integrity of the dataset, as a fully numeric, complete set of samples is required for the learning process. 

As stated in [1,2] our collection infrastructure includes data collectors, a collection server, and an Android monitoring application. Data is acquired for both Android events (SMS, WiFi, and Bluetooth) as well as Linux user-space (CPU load, memory load, and network statistics) and kernel-space metrics (performance counters). The collection server maintains the list of running Android applications (and correlation with corresponding process identification numbers) and based on application state manages monitoring via the data collectors. The Android application usually resides in background and allows high-level user interaction and control (including picking the infrastructure operating mode: idle, training, testing on a system-wide and per application level). Furthermore, part of the initial pre-processing of the dataset is that clearly anomalous readings are eliminated as data-points. This is partly achieved in the initial composition of the dataset, and partly done automatically when processing the numerical data.

The ground-truth target labels were manually set. Applications downloaded from reputable sources (top Google Applications) were marked as clean, while applications from the known malware sources (e.g., applications from the Malgenome set) were considered malware.

The dataset is first processed by parsing the feature data and identifying all the values associated with a unique application name on a particular feature set. These values are then averaged to obtain a single value for the {application name; feature} pair. The average value is obtained by running a first pass on the values and calculating their mean and variance, after which a new mean is computed, taking into consideration only the values that fall within a 95% confidence interval from the natural mean. In the extreme case that no values fall within this range, the median value of the set is selected instead. This contributes to the rejection of extreme sensor values, which are thought to most likely be the result of erroneous momentary interrogation. 

Once all unique {application name; feature} values are parsed for a particular feature, the application names are checked against the existing list and only the pairs for which the application name already exists are kept. This is done to ensure the integrity of the dataset, in which all samples (i.e., applications) must have existing values for all features. The end result is a dataset with 361 samples for 19 features, plus the target labels, which are the inputs to the learning algorithms. 

The features are all numeric, so there is no need to apply any type of coding for categorical variables. The target class labels are either clean (coded to 0) or malware (coded to 1).

The final dataset has its features normalized. The value for each sample within a feature is updated by subtracting the mean ($\mu_{j}$) and dividing by the standard deviation ($s_{j}$) of that feature (*j*), as shown below.

(1) $$x_{ij}:=\frac{x_{ij}-\mu_{j}}{s_{j}}$$

This ensures that all input features are of similar range and centered on zero, which helps the speed and convergence of the learning process, as well as removing potential biases caused by the varying magnitudes. A cross-section of the dataset is exemplified in Table 4. Notice that the unique application names have been removed and each sample is now uniquely identifiable only implicitly through its row number. The actual data used for training is structured with samples as rows and features as columns, but is shown here transposed, for readability. It also includes an intercept term, which is a column of 1-s, not shown here. The sample and feature labels are not present in the actual dataset and are shown in the table only for convenience.

The dataset is then split into three sets, for training, cross-validation, and testing. The training set is used to learn the optimized parameters of the prediction model, the cross-validation set is used to tune its meta-parameters, and the testing set is used exclusively to rate the model performance on data it had not previously seen. The parameters and meta-parameters differ for each learning model and are explained in their respective sections. 

The split is done randomly each time the overall script starts, so the data is different for each complete run which learns and evaluates the three models, meaning the overall results differ slightly at each run. However, the same training, cross-validation, and test sets are used for learning and evaluation on each of the three learning models, within a complete run of the script, so that their performance can be properly compared. 

The samples are split into 70% for the training set, 15% for the validation set and 15% for testing. This is done by generating a random index with the same length as the number of samples and then taking the appropriate percentage splits, using the index as the row number inside the dataset. In the context of 361 total samples, this works out to 253 samples for training, 54 samples for validation, and 54 samples for testing. 

A True Positive (TP) is a sample correctly identified as malware, a True Negative (TN) is a sample correctly classified as clean, a False Positive (FP) is a clean sample misclassified as malware, and a False Negative (FN) is a malware sample misclassified as clean. A chart showing the number and rates of samples thus classified is called a Confusion Matrix. The results of each algorithm include such a chart for each sample set and for the overall set. 

Two standard loss functions are used for the classification algorithms, Cross-Entropy Loss and Hinge Loss. Cross-entropy is the default loss function used for binary classification problems. It is intended for use with binary classification where the target values are in the set {0, 1}, as is the default coding in our dataset. It is used for the Logistic Regression and Pattern net learning algorithms [24]. Using Cross-Entropy Loss, the cost function to be minimized is shown below. $h_{\theta }\left(x^{i}\right)$ is the hypothesis outputted by the model (see also Equation (5)) and $y^{i}$ is the ground-truth label for that sample.

(2) $$J\left(\theta \right)=\frac{1}{m}\overset{m}{\underset{i=1}{\sum }}[y^{i}\left(-logh_{\theta }\left(x^{i}\right)\right)+\left(1-y^{i}\right)\left(-\log\left(1-h_{\theta }\left(x^{i}\right)\right)\right)]+\frac{\lambda }{2m}\overset{n}{\underset{j=1}{\sum }}\theta_{j}^{2}$$

where $h_{\theta }\left(x^{i}\right)=\frac{1}{1+e^{-\theta^{T}x^{i}}}$.

Hinge Loss is an alternative to cross-entropy for binary classification problems, primarily developed for use with SVM models. It is intended for use with binary classification where the target values are in the set {−1, 1}, which are set automatically by the learning algorithm. The Hinge Loss function encourages examples to have the correct sign, assigning more error when there is a difference in the sign between the actual and predicted class values [24]. The formula of the Hinge Loss function is shown below.

(3) $$J\left(\theta \right)=C\overset{m}{\underset{i=1}{\sum }}\left[y^{i}cost_{1}\left(\theta^{T}x^{i}\right)+\left(1-y^{i}\right)cost_{0}\left(\theta^{T}x^{i}\right)\right]+\frac{1}{2}\overset{n}{\underset{j=1}{\sum }}\theta_{j}^{2}$$

where $cost\left(x\right)=\{\begin{array}{c} 0,\\ 1-y*k\left(x\right), \end{array}\begin{array}{c} y*k\left(x\right)\geq 1\\ else \end{array}$, k(x) being the similarity (kernel) function [25,26,27].

The following metrics are used for evaluation, as shown in Table 5.

Accuracy is the rate of correctly classified instances, irrespective of class. Precision is the fraction of relevant instances among the retrieved instances, i.e., the number of correctly predicted positives out of the samples that were predicted positive. Recall is the fraction of total relevant instances retrieved, i.e., the number of correctly predicted positives out of the samples that actually were positive. The F1 score is the harmonic mean of precision and recall [28]. 

Table 6 below gives an overview of the parameters and criteria used for optimization at each level of the training process. 

Logistic Regression is used to obtain a model for the classification and prediction of a binary outcome from a set of samples, in the presence of more than one explanatory variable. The procedure is quite similar to multiple linear regression, with the exception that the response variable is binomial [29,30].

The optimization problem is described as $\hat{Y}=\theta *X$, where *X* is the input data, namely the dependent variables, containing all data-points, plus an intercept term, which helps prevent overfitting. *Y* is the output data that the algorithm is trying to learn, and θ is the matrix of coefficients used to estimate *Y* from *X*. The challenge is finding the best coefficients, which minimizes the error between the actual *Y* and the estimate. This is obtained from $\hat{Y}=\theta_{LR}*X$, or, in extended form:

(4) $$\left[\begin{array}{c} \hat{y_{1}}\\ \hat{y_{2}}\\ \begin{array}{c} \hat{y_{3}}\\ \vdots \\ \hat{y_{n}} \end{array} \end{array}\right]=\left[\begin{array}{ccc} \theta_{11} & \cdots & \theta_{1\left(m+1\right)}\\ \vdots & \ddots & \vdots \\ \theta_{n1} & \cdots & \theta_{n\left(m+1\right)} \end{array}\right]\left[\begin{array}{c} x_{1}\\ x_{2}\\ \begin{array}{c} \vdots \\ x_{m}\\ int \end{array} \end{array}\right]$$

Once the best available set of coefficients (*θ*) is found, a prediction for a given sample is done using the function:

(5) $$p\left(x\right)=\{\begin{array}{c} 0,\\ 1, \end{array}\begin{array}{c} h_{\theta }\left(x\right)<th\\ h_{\theta }\left(x\right)\geq th \end{array}$$

where $h_{\theta }\left(x\right)=\frac{1}{1+e^{-\theta^{T}x}}$ and *th* is the prediction threshold.

**Figure 2:**
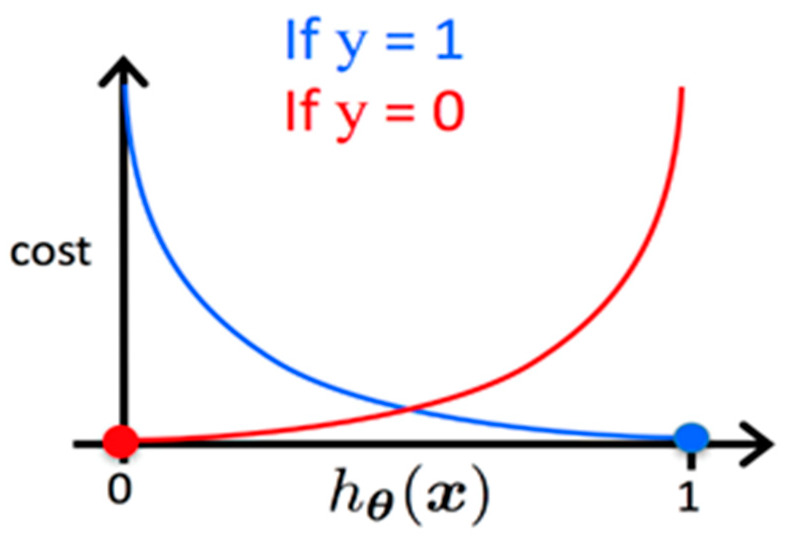
Logistic Regression cost function.

The cost function, shown previously in Equation (2), is computed as a measure of the difference between estimated values and the ground truth for the input data. A visualization of the cost for a particular sample is shown in Figure 2, where *y* is the ground truth and $h_{\theta }\left(x\right)$ is the hypothesis outputted by our model [25]. 

If there are too many features, the learned hypothesis may fit the training set very well, but fail to generalize to new examples, which is called overfitting [26]. Conversely, if there are not enough representative features or if the number of the training examples is not large enough to correctly create the model, it would underfit. Parameters such as alpha (α), the learning rate, or lambda (λ), the regularization parameter, must also be taken into account. The learning rate, α, should not be too large, because it could lead the algorithm to diverge, yet a value too small would cause slow learning and the cost function can get stuck in local minima [31]. Faster gradient descent can be obtained via an adaptive learning rate. Lambda (λ) is the regularization parameter and it determines how strongly a model is penalized for fitting too closely the learned features on the available data [25].

**Figure 3:**
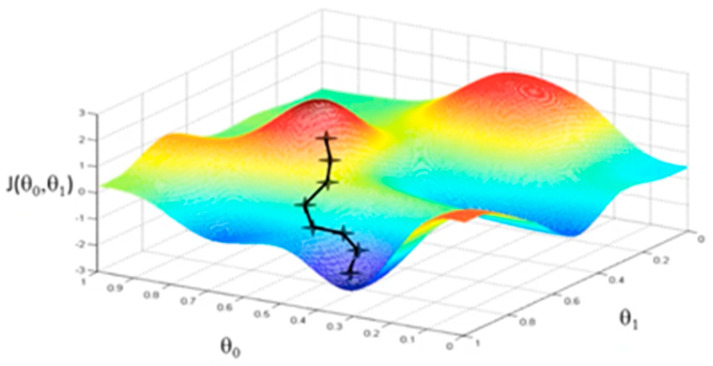
Gradient Descent algorithm.

Gradient descent is an optimization algorithm where the potential solution is improved each iteration by moving along the feature gradient in the variable space. While it requires that the target function be differentiable and it is somewhat susceptible to local minima, gradient descent provides a stable and computationally inexpensive algorithm for function optimization. Figure 3 shows a visualization of the Gradient Descent algorithm, for two thetas [25]. This would correspond to a one variable learning problem, for which $\theta_{1}$ is the coefficient of the independent variable x, and $\theta_{0}$ is the intercept term. $J\left(\theta_{0},\theta_{1}\right)$ is the associated cost for each tuple of the two theta parameters, shown as the landscape on which the gradient descent algorithm searches for optima.

SVMs, also called Large Margin Classifiers, are supervised learning models used for classification and regression. The input data is classified by optimizing the hyperplane equation so that the distance to the data representing the classes is maximum [32]. In addition to performing linear classification, SVMs can efficiently perform a non-linear classification using kernels, implicitly mapping their inputs into high-dimensional feature spaces [25].

**Figure 4:**
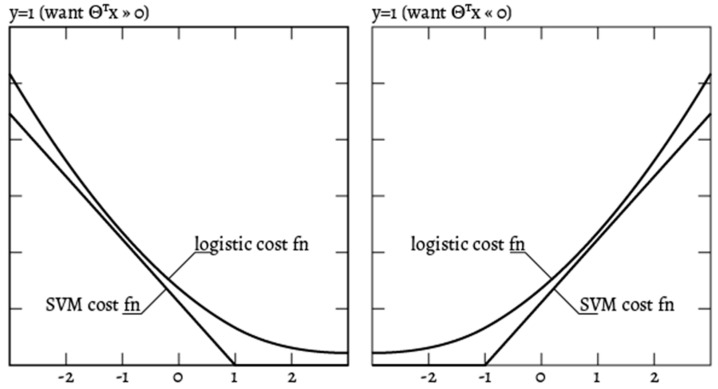
SVM and Logistic Regression cost functions.

Having the training data, the problem is to determine the parameters of the hyperplane that best separates the data into classes. The cost function for SVMs was shown previously in Equation (3). The parameter C is important for the system because it is responsible for finding the minimum of the cost function, as well as providing regularization. If the value of C is too large then the SVM will change the decision boundary in an intent to integrate the outliers which produces overfitting, but if C is too small, there is a risk of high bias [25]. The similarity function, named kernel, is used by the SVM to draw the decision boundary. Kernels must be semi-positive and symmetrical. The paper investigates the use of three of the most common options: linear, polynomial, and Gaussian (Radial Basis Function), in order to find normally distributed features across the feature map, while also being able to find features that have unusual values [33]. Because of faster execution, an SVM with a Gaussian kernel might outperform Logistic Regression for a medium-sized dataset. SVM finds a solution hyperplane which is as far as possible for the two categories while Logistic Regression does not have this property. In addition, Logistic Regression is more sensitive to outliers than SVM, because the Logistic cost function diverges faster than Hinge Loss [33]. Figure 4 provides a visual representation of the difference between the two cost functions, using the notations of *y* and $\theta^{T}x$ discussed earlier in the paper [34].

**Figure 5:**
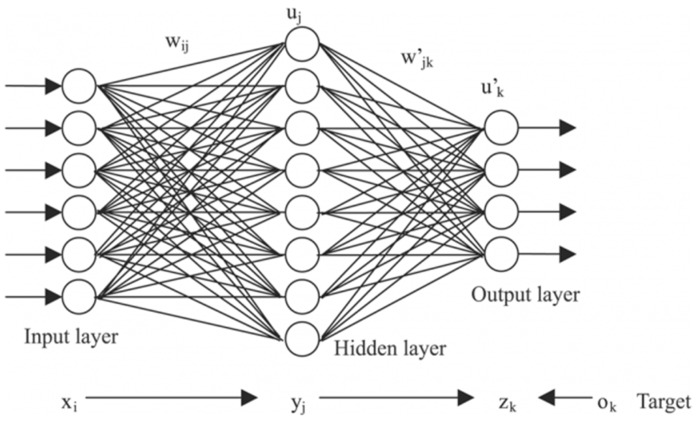
Artificial neural network with one hidden layer.

Artificial neural network models are based on a layer of hidden features (i.e., neurons), which replace the specific features used in other models, and which control the classification. Neural networks have an input layer that matches the dimension of an input sample and an output layer which matches the target variable. The model is optimized by successively tuning the weights of these neurons, through a process called back-propagation. A standard neural network is exemplified in Figure 5 [35,36], where $x^{i}$ are the features of the input layer, i.e., the dataset features shown previously, $w_{ij}$ are the weights associated with the transition of from the input layer to the hidden layer, $u_{j}$ are the artificial features of the hidden layer, the number of which is an important meta-parameter, to be chosen by the designer, $w_{jk}^{\prime }$ are the weights associated with the transition from the hidden layer to the output layer, and $u_{k}^{\prime }$ are the output features, or dependent variables, of which there is only one (*y*) in the case of binary classification, such as presented in this paper.

## 3. Results

### 3.1. Logistic Regression

The first learning algorithm investigated is Logistic Regression with polynomial features. The initial dataset is expanded by mapping the second- and third-degree polynomials of the original features, to allow the algorithm to better account for the nonlinearities in the dataset. This means that:

(6) $$X:=\left[X\;X^{2}X^{3}\right]$$

for all training, cross-validation, and test sets. There are 57 resulting features, plus an intercept term (see also Equation (4)), used to model all other phenomena on which the target might be dependent, which also helps prevent overfitting. This comes out to 58 total features, training on 253 samples. The polynomial degree was chosen to provide a good equilibrium between accounting for nonlinearities and the risk of overfitting the dataset.

**Figure 6:**
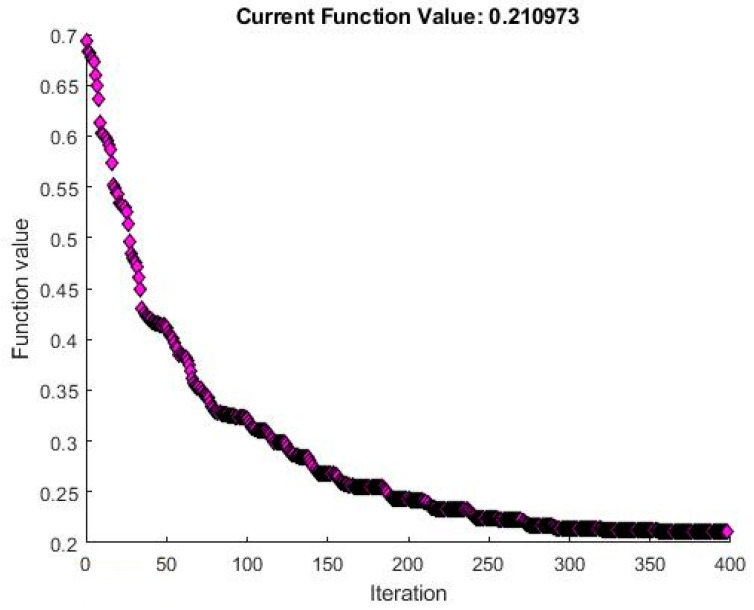
Logistic Regression Cost Function over algorithm iterations.

One training run of the algorithm takes place for up to 400 iterations, starting from an initial parameter vector (*θ*) of all zeroes. The decrease of the cost function in an example run using gradient descent is shown in Figure 6.

Cross-validation is done simultaneously for two meta-parameters, lambda (λ) and the prediction threshold (th). The ranges of values for the two meta-parameters are shown in Table 7.

There are therefore 40 combinations of possible {lambda; threshold} parameters. Each of these is used to perform a training run, for which the theta parameter vector is stored, and the F1 score of each resulting model is evaluated, with the best model being kept as the overall Logistic Regression model. Table 8 shows an example of the results obtained for a complete suite of training runs using Logistic Regression. For brevity, results are shown for each lambda, but with the threshold level set at the chosen best.

An alternative approach was explored, whereby cross-validation was done separately, first for the lambda (*λ*) parameter, using the evaluation of the cost function. The best lambda (*λ*) and its respective theta parameter vector were chosen, after which the best prediction threshold was evaluated based on the F1 score. However, this approach consistently obtained poorer results in terms of F1 score to the simultaneous cross-validation method mentioned earlier.

As can be seen from Table 8, the best F1 score on the validation set is 0.89, which leads to the choice of λ=10 and th=0.5. Depending on the initial dataset split, which is random, the results do have a slight variation, both in the actual best meta-parameters chosen, as well as the resulting best F1 score for the validation set. The results obtained in Table 8 are among the best recorded for Logistic Regression, after multiple runs, with the evaluation score on the validation set being about as high as can be expected. The final evaluation for the Logistic Regression algorithm is the F1 score of 0.84 recorded on the test set.

**Figure 7:**
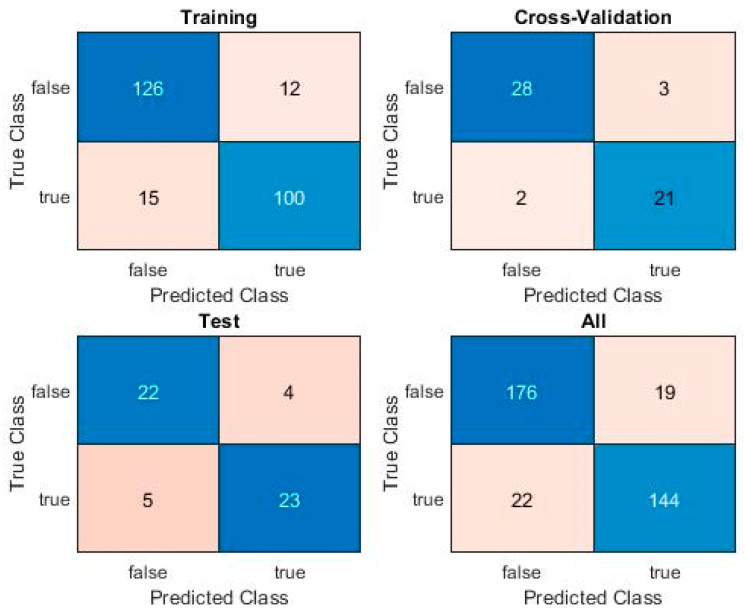
Confusion Matrix for Logistic Regression.

The Confusion Matrix for the chosen Logistic Regression model is shown in Figure 7, displaying the Confusion Matrices for each of the training, cross-validation, and test sets, as well as the overall Confusion Matrix, for the entire dataset.

### 3.2. Shallow Neural Network for Pattern Recognition

**Figure 8:**
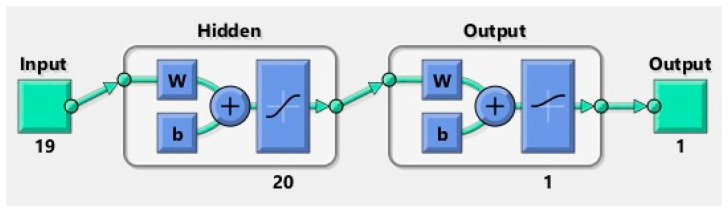
PatternNet architecture.

The following model is a shallow pattern recognition neural network (PatternNet), with a single hidden layer composed of 20 neurons. The indices for the training, cross-validation, and test sets were kept from the initial dataset split and provided to the pattern net, so that the same sets are used for training and testing, which allows a proper comparison of the results obtained by the models. Figure 8 shows the architecture of a PatternNet neural network, with w being the transition weights of the network and b being the weights of the bias, i.e., intercept, term, which is automatically added by the network upon initialization.

**Figure 9:**
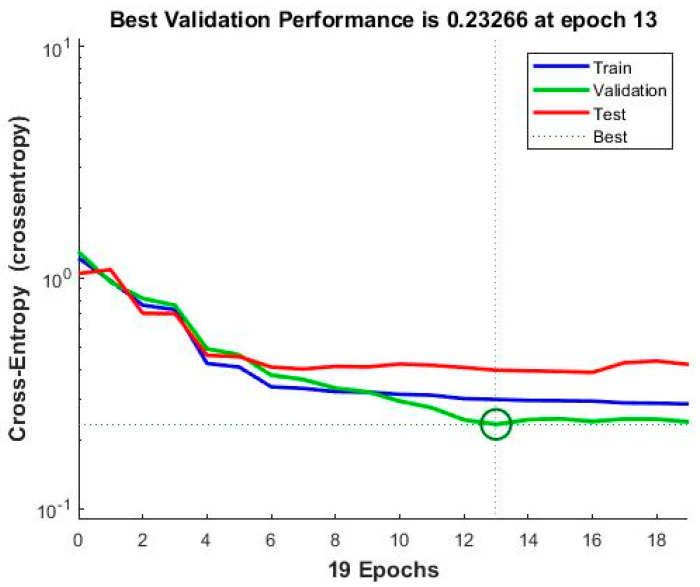
PatternNet performance.

Training is performed using Scaled Conjugate Gradient, with a similar Cross-Entropy Loss function as the Logistic Regression. The point of cross-validation, in this case, is to supervise the performance of the network and prevent overfitting. Figure 9 shows the performance of the network on the three sets, with the set of weights chosen for best performance on the validation set, displaying the loss function value throughout the training iterations, i.e., epochs, for the training, cross-validation, and test sets.

The results of the PatternNet model are shown in Table 9.

**Figure 10:**
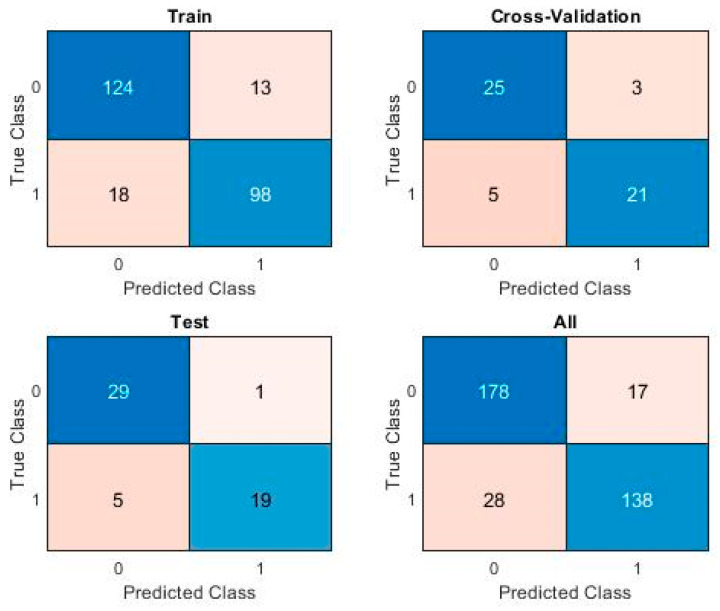
PatternNet Confusion Matrix.

After multiple runs, the best F1 score obtained by a PatternNet model on this challenging dataset is 0.86. The associated Confusion Matrix is shown in Figure 10 for each of the training, cross-validation, and test sets, as well as the overall Confusion Matrix, for the entire dataset.

### 3.3. Support Vector Machines

The SVM algorithm is trained using the same dataset split as for the other two models. Cross-validation is done on the choice of kernel, with the three possible hardcoded options of $K={\{}^{\prime }linear^{\prime }{,}^{\prime }gaussian^{\prime }{,}^{\prime }polynomial{\left(3\right)}^{\prime }\}$. The maximum degree of the polynomial kernel was chosen to be comparable to the maximum degree of the feature mapping from Logistic Regression. An SVM model is trained for each of these kernel options and the best one is chosen, based on their respective F1 scores. The results are shown below in Table 10.

**Figure 11:**
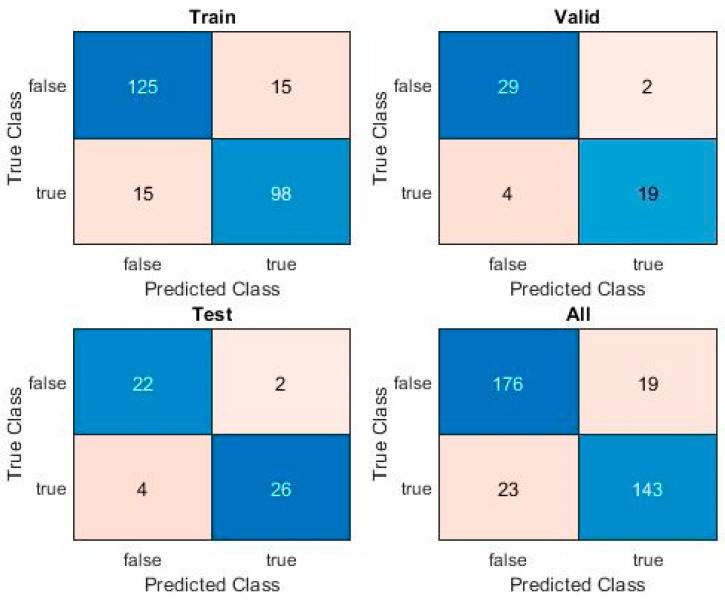
SVM Confusion Matrix.

The associated Confusion Matrix is shown in Figure 11 below, for each of the training, cross-validation, and test sets, as well as the overall Confusion Matrix, for the entire dataset.

## 4. Discussion

Table 11 provides an overall comparison of obtained metrics on the training, validation, and test sets, for each proposed model, using the meta-parameter values selected during validation. The results show that all of the three investigated algorithms perform reasonably well on a challenging dataset, with SVMs having an edge in performance on the test set, as well as maintaining a good equilibrium between the training, cross-validation, and test results. 

A Cochran’s Q test [37,38] was performed to evaluate the results, with the null hypothesis being that the three algorithms have similar performance in classification. The test obtains a *p*-value of $5.7268*10^{-10}$ and a Cochran Q value of 42.5614, for which the null hypothesis can be safely rejected at the α=0.05 significance level, providing proof that the SVM model significantly outperforms the others.

Furthermore, a comparative post-hoc analysis of the statistical significance of the results obtained through the three models was performed using three McNemar tests [39,40] for each of the three pairs of models. The tests are undertaken on pairs of models, since a McNemar Test can only be run on two models at a time. The first test (exact–conditional McNemar) compares the accuracies of the two models, while the second (Asymptotic McNemar) and third (Mid-*p*-value McNemar) tests assess whether one model classifies better than the other. The null hypothesis for the first test is that the two models have equal predictive accuracies. The null hypothesis for the second and third tests is that one of the models is significantly less accurate than the other. The comparison is not cost sensitive, i.e., it assigns the same penalty for different types of misclassification. Detailed descriptions of McNemar tests and the procedure used can be seen in [40,41]. Table 12 shows the results of the statistical significance tests, where h is the hypothesis test result (h=1 indicates the rejection of the null hypothesis, while h=0 indicates a failure to reject the null hypothesis), p is the *p*-value of the test, and e1 and e2 are the classification losses or misclassification rates of the respective two models (which are the same, irrespective of test).

The comparison between Logistic Regression and PatternNet is somewhat inconclusive. While the exact–conditional test cannot reject the null hypothesis, that the two methods have similar accuracies, neither can the other two tests reject the assumption that one test performs better than the other, although it should be noted that the *p*-value of the first test is twice as large as the values for the second and third tests. However, in the comparisons with the SVM model, both for Logistic Regression, as well as for PatternNet, the null hypothesis of the first test is rejected, meaning that the two methods have dissimilar accuracies. This is further collaborated by the second and third tests, where there is strong evidence that the null hypothesis cannot be rejected, meaning that the SVM model significantly outperforms both the Logistic Regression, as well as the PatternNet, models.

The obtained *p*-values of the exact–conditional McNemar tests are manually corrected using the Discrete Bonferroni–Holm Method for Exact McNemar tests [42,43,44]. As stated by Holm (1979) [42], “except in trivial non-interesting cases the sequentially rejective Bonferroni test has strictly larger probability of rejecting false hypotheses and thus it ought to replace the classical Bonferroni test at all instants where the latter usually is applied”. Table 13 shows the corrected *p*-values and the resulting decision on the significance of the test.

The corrected *p*-values are obtained as described in Westfall et al. [43] and implemented in [44]. For the test result to be considered significant, its corrected *p*-value should be below the α=0.05 threshold. The first test is evaluated as expected, since the initial series of McNemar tests showed that there is no statistical difference in the performance of Logistic Regression and PatternNet, by a very high *p*-value, in relation to the threshold. The second test is most interesting, as the hypothesis that the Logistic Regression model and the SVM model have significantly different performance is now rejected, upon strengthening the test. However, it should be noted that it is a borderline decision, and the test would have been accepted at a α=0.1 significance level. The third test reinforces the conclusion that the SVM model performs significantly better, this time compared to the PatternNet model, meeting the significance requirement by a wide margin.

However, all their performances are satisfactory and comparable on the initial metric used. The intention is that the dataset be expanded in the future, at which time further testing will be undertaken to evaluate the ability of each model to generalize, as well as the performance of various ensemble methods, possibly comprising two or more of these same models.

The literature review has yielded few comparable studies for the chosen methods on smartphone applications, without even taking into account the considerably different datasets, features, extraction techniques, etc. that other studies work with. Most papers discuss detection accuracy, with very few using the F1 score metric. The reported accuracy also varies considerably across methods and especially datasets. The final metric values, on the test set, for the linear kernel SVM, compare favorably with most papers in the literature review, for which similar metrics were available. The closest comparison is the application described in [2], which obtains slightly higher metrics on a previous version of the dataset. 

While the final hard numbers of the F1 score metric and the statistical significance tests clearly favor the SVM implementation, the difference in performance is oftentimes a relatively small number of misclassified samples, which is one of the issues to keep in mind when working with a small number of data-points.

Therefore, one of the main directions for future research is expanding the dataset, both in terms of the number of data-points (samples), as well as the number of features, where applicable. This would be a great benefit to the ability to train and test the models, as well as allow the investigation of deeper and more complex model architectures, some of which were touched upon in the discussion on the state of the art. 

Another interesting future option is ensemble learning, which leverages the positive results of having three good classifiers, whose outputs can then be composed into a final prediction. This is particularly appealing, given the comparatively small difference in accuracies of the three investigated models.

In addition, detection of anomalous behavior from the user’s interaction pattern standpoints could be a promising topic for further research, starting from the framework already described.

The obvious future step for the application is that of actual hardware implementation on a working prototype, through which future data can also be more easily gathered. The dataset split procedure, which was discussed at length throughout the paper, should give the model a good ability to generalize to as yet unseen data. 

## Figures and Tables

**Table 1:** Measurable events and description.

Event	Intercepted Information
SMS	an SMS is sent, destination phone number, message content
Call	outgoing phone call takes place, destination phone number
WiFi	Wi-Fi state—enabled or not
Bluetooth	Bluetooth state—enabled or not
App Install	package installation/uninstallation and package name
Sensors	value information for registered sensors
Camera	Camera state—on or off
NFC	NFC state—on or off
Activity	current state of the Activity: Create, Start, Stop, Pause, Resume, Destroy
Runtime Crash	a runtime crash of the application is happening
ANR	Application Not Responding dialog box is being generated

**Table 2:** Example of raw data obtained from software sensors.

Application Name	sent_bytes_wifi_fore	Application Name	sent_bytes_wifi_fore
air.CandyCatcher	23461	air.com.innmenu.free	7293
air.CandyCatcher	23617	air.com.innmenu.free	9316
air.CandyCatcher	23825	air.com.innmenu.free	12354
air.CandyCatcher	23825	air.com.innmenu.free	13367
air.CandyCatcher	23825	air.com.innmenu.free	19440
air.com.innmenu.free	7293	air.com.innmenu.free	21469

**Table 3:** Description of sensor data features.

No.	Feature Name	Description
1	SysCycles_subset	System-wise CPU cycles collected via performance counters
2	Cycles_subset	Application CPU cycles collected via performance counters
3	Load1_subset	CPU load for 1 min collected via procfs (cat /proc/loadavg) in user-space
4	Load5_subset	CPU load for 5 min collected via procfs (cat /proc/loadavg) in user-space
5	Load15_subset	CPU load for 15 min collected via procfs (cat /proc/loadavg) in user-space
6	Total_occ_mem_subset	Total memory information collected via procfs (cat /proc/meminfo) in user-space
7	Memory_subset	VmSize memory information collected via procfs (cat /proc/$pid/status | grep VmSize) in user-space
8	RSS_memory_subset	VmRSS memory information collected via procfs (cat /proc/$pid/status | grep VmRSS) in user-space
9	Threads_subset	Number of threads collected via procfs (cat /proc/$pid/status | grep Threads) in user-space
10	Recv_bytes_wifi_back_subset	Number of bytes, obtained using AOSP events and procfs stats, received via WiFi while the application is in background
11	Recv_packets_wifi_back_subset	Number of packets, obtained using AOSP events and procfs stats, received via WiFi while the application is in background
12	Sent_bytes_wifi_back_subset	Number of bytes, obtained using AOSP events and procfs stats, sent via WiFi while the application is in background
13	Sent_packets_wifi_back_subset	Number of packets, obtained using AOSP events and procfs stats, sent via WiFi while the application is in background
14	Recv_bytes_wifi_fore_subset	Number of bytes, obtained using AOSP events and procfs stats, received via WiFi while the application is in foreground
15	Recv_packets_wifi_fore_subset	Number of packets, obtained using AOSP events and procfs stats, received via WiFi while the application is in foreground
16	Sent_bytes_wifi_fore_subset	Number of bytes, obtained using AOSP events and procfs stats, sent via WiFi while the application is in foreground
17	Sent_packets_wifi_fore_subset	Number of packets, obtained using AOSP events and procfs stats, sent via WiFi while the application is in foreground
18	CPU_usage_user_float_subset	User-space CPU usage percentage
19	CPU_usage_system_float_subset	Total CPU usage percentage

**Table 4:** Example of dataset samples.

	App 1	App 2	App 3	App 4	App 5	App 6	App 7	App 8	App 9
Feat 1	2.1260	−0.2039	−0.9102	−1.0380	−0.7869	−0.4426	−0.5818	2.0124	0.3142
Feat 2	1.5776	0.2069	−0.8571	−0.8805	−0.7298	−0.8488	−0.6119	4.5156	−0.2921
Feat 3	1.3070	1.5609	−0.9932	−0.7135	0.8064	−1.4613	−0.6617	−0.0143	1.9401
Feat 4	2.0614	0.5280	−0.5014	−0.3219	0.4668	−0.7027	−0.2998	−0.3167	0.6111
Feat 5	4.8613	−0.0313	−0.4700	−0.3211	1.4666	−0.3339	−0.4885	−0.2823	0.1868
Feat 6	1.6025	−0.2129	−0.4023	0.7248	1.3224	−0.9621	−1.3607	1.0606	0.5359
Feat 7	1.6277	1.7521	−1.1560	−1.0896	0.8968	−1.1018	−1.1545	−0.7817	0.3637
Feat 8	1.3134	1.5573	−1.0430	−0.9086	−0.0139	−0.9776	−0.9513	−0.9219	0.0963
Feat 9	0.9160	2.1956	−1.2540	−1.1672	0.9956	−1.0804	−1.0804	−0.8200	0.3952
Feat 10	−0.1558	−0.1579	−0.1550	−0.1579	−0.0435	−0.1579	−0.1579	−0.0612	−0.1579
Feat 11	−0.1674	−0.1841	−0.1590	−0.1841	0.0255	−0.1841	−0.1841	0.0255	−0.1841
Feat 12	−0.2925	−0.3570	−0.3128	−0.3803	0.6953	−0.3803	−0.3803	0.5122	−0.3803
Feat 13	−0.2251	−0.2320	−0.2182	−0.2667	0.2182	−0.2667	−0.2667	0.3775	−0.2667
Feat 14	−0.1042	−0.1742	−0.2019	−0.1976	−0.1635	−0.2020	0.3298	−0.1763	−0.1719
Feat 15	−0.1062	−0.1707	−0.2224	−0.1955	−0.1546	−0.2245	0.2975	−0.1638	−0.1750
Feat 16	0.0372	−0.1001	−0.4477	−0.2964	0.1316	−0.4582	−0.0927	0.0206	0.1077
Feat 17	−0.0349	−0.2000	−0.3626	−0.2479	−0.1387	−0.3678	0.2105	−0.0466	−0.2137
Feat 18	−0.0548	−0.0547	−0.0202	−0.0548	−0.0548	0.0555	−0.0548	−0.0528	−0.0548
Feat 19	−0.0660	−0.0659	−0.0323	−0.0660	−0.0660	0.0497	−0.0660	0.0804	−0.0660

**Table 5:** Classifier metrics.

Number of True Positives	*TP*
Number of True Negatives	*TN*
Number of False Positives	*FP*
Number of False Negatives	*FN*
Accuracy	$ACC=\frac{TP+TN}{TP+TN+FP+FN}$
Precision (Positive Predictive Value—PPR)	$PPR=\frac{TP}{TP+FP}$
Recall (True Positive Rate—TPR)	$TPR=\frac{TP}{TP+FN}$
F1 Score	$F1=2*\frac{PPR*TPR}{PPR+TPR}=2*\frac{precision*recall}{precision+recall}$

**Table 6:** Parameters, meta-parameters and criteria.

	Training		Cross-Validation		Testing
	Optimize	Evaluate	Choose/Test	Evaluate	Evaluate
Logistic Regression	Theta	Cross-Entropy	Lambda	F1 score	F1 score
			Threshold		
Support Vector Machine	Margin	Hinge	Kernel	F1 score	F1 score
Pattern net	Weights	Cross-Entropy	Weights	Cross-Entropy	F1 score

**Table 7:** Ranges of values for lambda and threshold.

Lambda	0.1	0.3	1	3	10	25	50	100
Threshold	0.1	0.3	0.5	0.7	0.9			

**Table 8:** Logistic Regression results on varying lambda.

λ	Training Set				Cross-Validation Set				Test Set			
	Acc	Prec	Rec	*F1*	Acc	Prec	Rec	*F1*	Acc	Prec	Rec	*F1*
0.1	0.91	0.92	0.87	0.89	0.87	0.83	0.87	0.85	0.81	0.80	0.86	0.83
0.3	0.91	0.91	0.88	0.89	0.89	0.87	0.87	0.87	0.83	0.83	0.86	0.84
1	0.90	0.90	0.88	0.89	0.89	0.87	0.87	0.87	0.83	0.83	0.86	0.84
3	0.90	0.90	0.87	0.88	0.89	0.87	0.87	0.87	0.83	0.83	0.86	0.84
10	0.89	0.89	0.87	0.88	0.91	0.88	0.91	0.89	0.83	0.85	0.82	0.84
25	0.89	0.89	0.85	0.87	0.89	0.87	0.87	0.87	0.81	0.82	0.82	0.82
50	0.88	0.88	0.84	0.86	0.89	0.87	0.87	0.87	0.80	0.79	0.82	0.81
100	0.88	0.88	0.84	0.86	0.87	0.83	0.87	0.85	0.80	0.79	0.82	0.81

**Table 9:** PatternNet results.

PatternNet	Accuracy	Precision	Recall	F1 Score
Training Set	0.88	0.88	0.84	0.86
Cross-Validation Set	0.85	0.88	0.81	0.84
Test Set	0.89	0.95	0.79	0.86

**Table 10:** SVM results on varying the kernel.

Kernel	Training Set				Cross-Validation Set				Test Set			
	Acc	Prec	Rec	*F1*	Acc	Prec	Rec	*F1*	Acc	Prec	Rec	*F1*
Linear	0.88	0.87	0.87	0.87	0.89	0.90	0.83	0.86	0.89	0.93	0.87	0.90
Gaussian	0.98	0.98	0.97	0.98	0.80	0.93	0.57	0.70	0.83	1.00	0.70	0.82
Polynomial	1.00	1.00	1.00	1.00	0.81	0.78	0.78	0.78	0.85	0.92	0.80	0.86

**Table 11:** Overall comparison of model results.

	Logistic Regression	PatternNet	SVM
	λ=10	20 Neurons	Linear Kernel
Training Set			
Accuracy	0.89	0.88	0.88
Precision	0.89	0.88	0.87
Recall	0.87	0.84	0.87
F1 score	0.88	0.86	0.87
Cross-Validation Set			
Accuracy	0.91	0.85	0.89
Precision	0.88	0.88	0.90
Recall	0.91	0.81	0.83
F1 score	0.89	0.84	0.86
Test Set			
Accuracy	0.83	0.89	0.89
Precision	0.85	0.95	0.93
Recall	0.82	0.79	0.87
F1 score	0.84	0.86	0.90

**Table 12:** McNemar tests for significance of model performance.

	Exact–Conditional	Mid *p*-Value	Asymptotic
Logistic Regression vs. PatternNet			
h	0	0	0
p	0.2295	0.1147	0.1103
e1	0.1108		
e2	0.1274		
Logistic Regression vs. Support Vector Machine			
h	1	0	0
p	0.0488	0.9755	0.9761
e1	0.1108		
e2	0.0720		
PatternNet vs. Support Vector Machine			
h	1	0	0
p	0.0041	0.9977	0.9976
e1	0.1274		
e2	0.0720		

**Table 13:** Corrected exact McNemar tests for significance of model performance.

	Test 1	Test 2	Test 3
	LogReg vs. PatternNet	LogReg vs. SVM	PatternNet vs. SVM
Corrected *p*-value	0.2200	0.0800	0.0120
Significance	0	0	1
